# Production preferences cannot be understood without reference to communication

**DOI:** 10.3389/fpsyg.2013.00230

**Published:** 2013-04-26

**Authors:** T. Florian Jaeger

**Affiliations:** Brain and Cognitive Sciences, Human Language Processing Lab, University of RochesterRochester, NY, USA

MacDonald (2013) proposes that comprehenders are sensitive to statistical patterns in their language input (Claim 1). These patterns are hypothesized to result from speakers' preferences in production, aggregated over the population (Claim 2). Production preferences are taken to be primarily determined by biases that serve production ease, thereby improving fluency (Claim 3). These three claims, together constituting the core of the PDC, are an ambitious endeavor to tie together several lines of research in psycholinguistics and linguistics. Here, I focus on the second and third claim, that it is predominantly “production ease,” rather than communicative pressures, that drives production preferences and hence language form (M, p. 13; cf. Bard et al., 2000; Ferreira and Dell, 2000; Arnold, 2008; Ferreira, 2008; Lam and Watson, 2010).

In contrast, I argue that production preferences and language form are unlikely to be understood without reference to communication. Specifically, production preferences are the result of at least two competing type of biases: biases toward production ease and biases toward ease, or at least *success*, of comprehension (Zipf, 1949). I refer to a weak version of the second type of bias as robust information transfer.^1^ Two hypotheses about *how* robust information transfer might affect production preferences are often conflated in the literature. First, speakers might continuously “estimate” their interlocutors' beliefs and structure their utterances based on these estimates. This claim, often referred to as audience design, is what production researchers (incl. M) tend to have in mind when they reject the idea that production preferences are affected by communicative biases. Many consider this claim implausible because production seems too demanding to allow additional computations (Ferreira, 2008). I share Tanenhaus's position that such intuitions are often misleading (Tanenhaus, 2013). Here, however, I pursue an alternative hypothesis, that communicative biases affect production preferences through learning and generalization across previous experiences (building on Jaeger and Ferreira, in press).

## Production ease is not enough

Speakers tend to lengthen words (*theeee*) or produce additional words, such as filled pauses (*uh, um*, etc.) or optional function words (e.g., *I think (that) you're right*), when upcoming material is not available for production (Fox Tree and Clark, 1997; Ferreira and Dell, 2000; Clark and Fox Tree, 2002). M claims that “[…] speakers in this situation attempt to gain extra planning time” (M, p. 5; Race and MacDonald, 2003). This raises an important question that ease-of-production accounts have so far failed to address: if speakers need more time, why do they not simply halt articulation until the next word is available? It would arguably be *less* effortful and less memory demanding to suspend speech, and continue without producing the additional words once the upcoming material is available. Indeed, the few studies that have addressed this question have found no evidence that the insertion of optional words actually helps to alleviate planning difficulty. To the contrary, filled pauses are more likely to be followed by speech suspension than expected by chance (Clark and Fox Tree, 2002). Similarly, the presence of optional *that* is associated with *lower* fluency following it, even after controlling for other factors known to affect fluency (Jaeger, 2005, section 3).

Another reason for the bias against speech suspension might be that speakers aim to avoid interruption by others (see references in Clark and Fox Tree, 2002, p. 90). First, it is worth noting that such an explanation would no longer appeal exclusively to production ease. Furthermore, this hypothesis, too, seems incompatible with existing evidence (Fox Tree and Clark, 1997, p. 165–176; Clark and Fox Tree, 2002, p. 90). For example, producing *theeee* rather than *the* is associated with a *higher* probability of being interrupted by interlocutors (Fox Tree and Clark, 1997). At the very least, this means that lengthening *the* is not sufficiently effective in increasing fluency.

One hypothesis I have entertained elsewhere is the “don't stop a running car” metaphor (e.g., Jaeger, 2010a): it is possible that speakers go through extra articulation effort in order to avoid speech suspension because it is easier to continue talking than to start again (e.g., because this allows speakers to benefit from statistical contingencies between linguistic units). Regardless of whether this hypothesis is correct, it is clearly premature to assume that only production ease can affect speakers' preferences.

## Making sense of production by keeping in mind *why* we speak

An alternative explanation comes from communication accounts (e.g., Clark and Fox Tree, 2002; Aylett and Turk, 2004; Jaeger, 2010b). Clark and Fox Tree (2002) propose that the additional material serves as a signal to comprehenders about the state of the speaker's production system. Here we propose that, in addition to production ease, production is affected by a bias for robust information transfer. One frequent reason for speaking is that we want to convey information (be it semantic, pragmatic, or social in nature). This bias often competes with production ease (Zipf, 1949). Conveniently, striking a balance between these two types of biases also tends to maximize the *rate* of information transfer (cf. Aylett and Turk, 2004; van Son and van Santen, 2005; Levy and Jaeger, 2007; Piantadosi et al., 2011).

Why then do we produce filled pauses or optional function words? I propose that doing so allows speakers to remain informative even when they encounter production difficulty. For example, optional *that* contains information about the upcoming structure. But even filled pauses and other disfluencies contain information about upcoming material (they shift the probability distributions over upcoming words toward word that would *a priori* have been less probable, Shriberg and Stolcke, 1996). Producing filled pauses or optional function words thus achieves two things: it lowers the information density of the next words (which, in the context of *a priori* unexpected material, is efficient) and it allows listeners to start processing (i.e., predicting) the next word while the speaker is still planning it (for evidence, see Arnold et al., 2007).

It seems as if speakers are biased toward providing as much as possible of the information necessary to successfully transmit their message while balancing production ease. This view makes interesting predictions about the choice between different ways to deal with the burden of production. For example, in environments compatible with different optional words (both easily available, e.g., *that* or *uh*), speakers should prefer the more informative (*that*) rather than the less informative (*uh*). Furthermore, if both words are produced (e.g., because additional delay is required), they should prefer to order the more informative first (*that uh*, rather than *uh that*; the word *that* reduces the entropy of next possible words more so than the word *uh*). Both predictions are supported by existing data (Jaeger, 2005, Table 1). Crucially, production ease makes the opposite prediction [the word *uh* is phonologically simpler and, if anything more frequently produced, than optional *that* (based on Switchboard counts, Penn Treebank release)].

Finally, there are a variety of production preferences that are unexpected under accounts that attribute production preferences exclusively to production ease, but are predicted if there is a bias for robust information transfer. For example, across languages of the world, speakers are more likely to omit optional material if it is redundant in its context (Resnik, 1996; Jaeger, 2006, 2010b; Lee, 2006; Kurumada and Jaeger, in press). For example, Resnik (1996) finds that speakers of English are more likely to omit grammatical objects when their content is recoverable given the verb (e.g., *I already ate (dinner) a few hours ago*). Similarly, speakers of Japanese tend to omit the optional case-marker—*o*, when the intended meaning of the sentence is probable given its referential properties (e.g., *The doctor treated the grandma*), compared to when the intended meaning is improbable (e.g., *The grandma treated the doctor*, Kurumada and Jaeger, in press).

In short, there is a considerable body of evidence that lacks explanation if production preferences are exclusively driven by production ease. Instead, production preferences also seem to reflect a bias for robust information transfer. How would such a bias come to affect production preferences? That is, what mechanism might give rise to the observed patterns in language production (see M, p. 12)?

## A proposal: learning to produce communicatively efficient language forms

One important aspect that has so far received relatively little attention in this context is the role of *learning* (though see Jaeger and Snider, 2013). Relatively little is known about the extent to which implicit learning affects production. As M points out, there is much to be learned from research on motor control, which has long recognized the importance of learning in planning motor movements. In a very influential approach, the ability to plan and execute motor movements efficiently depends crucially on learning (forward models, Jordan and Rumelhart, 1992; Wolpert, 1997). In these models, actors learn to adapt their motor plans based on the prediction error experienced in previous movements (i.e., the difference between what was expected to happen and what was actually observed). I share M's intuition that these or similar accounts might help to understand how speakers learn to handle the burdens of production (e.g., fluent sequentialization, Dell et al., 2008; see also Chang et al., 2006).

Research on motor control, I believe, also holds the key toward a mechanistic account of communicatively efficient language production. There is increasing evidence that the implicit learning processes operating during control are sensitive to the actor's goals (Trommershäuser et al., 2005; Liu and Todorov, 2007; Wei and Körding, 2009; Knill et al., 2011). For example, recent research on motor control has found more learning after task-relevant errors (Wei and Körding, 2009). This raises the question as to what the relevant task dimensions are for language production. To the extent that one important function of speaking is to convey information (rather than to just make sounds), it would be expected that speakers do integrate feedback about the success of their communications into future production plans (Jaeger and Ferreira, in press). This feedback presumably includes speakers' perception of their own productions as well as implicit and explicit feedback from their interlocutors (e.g., failure to show an expected reaction, signs of confusion, request's for clarification). I take these questions to be a productive venue for future work that will clarify the extent to which a bias for robust information transfer affects production (and how).

Little is known about the extent to which these aspects affect language production. There is, however, some tantalizing evidence. In perturbation studies, speakers' productions are manipulated in real-time, leading to the (mis)perception of acoustic or phonological errors. This in turn leads speakers to adapt their productions, so as to compensate for the perceived error. Crucially, speakers adapt their productions in auditory perceptual, rather than motor, space (Guenther et al., 1998; Villacorta et al., 2007). Similarly, Frank (2011) finds that perturbation leads to stronger corrective adaptation if the (wrongly) perceived production would otherwise be confusable with existing phonological neighbors. These results suggest that adaptation in articulation is at least partially driven by prediction errors related to the likelihood of successful information transfer.

Researchers have just begun to investigate similar questions for language production beyond articulation. For example, speakers learn to avoid temporary syntactic ambiguities if they receive implicit feedback that communication failed (Roche et al., 2013). Further investigations of this type will help clarify the extent to which a bias for robust information transfer affects production (and, if so, how).

## Conclusion

The PDC presents an ambitious framework, tying together insights from production, comprehension, and typology. In particular, the link between production and comprehension has proven a powerful framework that guides our understanding of language processing. Yet, when it was first proposed (MacDonald et al., 1994), it was met with much incredulity. Perhaps one reason for this was that many thought the computations necessary to build expectations too complex. Research over the last two decades has shown that considerations about what is complex for the human brain can be misleading. With the benefit of hindsight, we can now say that the original formulation of this claim was, if anything, too timid. In addition to countless studies that have reported expectation-based effects on sentence processing, recent work suggests that comprehenders continuously adapt their beliefs about the statistics of the current linguistic environment (Wells et al., 2009; Farmer et al., 2011; Kamide, 2012; Jaeger and Snider, 2013; Fine et al., submitted). That is, the systems underlying language comprehension seem to be subject to automatic or near-automatic implicit learning (see also Farmer et al., in press).

I propose that we should avoid the mistakes of the past. Yes, language production and, in particular, sequentialization is complex (M, pp. 4, 14). This does not, however, imply that production preferences can be understood without reference to communication. This implication would be at odds with existing evidence from both language production (see references above) and language form (see Piantadosi et al., 2011, 2012). This caveat does not argue against the PDC. It does, however, show that solely focusing on production ease is problematic. If we, on the other hand, recognize that language is typically used to convey information and that the cognitive systems underlying language production seek to minimize variance along this task dimension, many otherwise puzzling properties of language production and language form have an explanation. In short, I propose that speakers, like comprehenders, implicitly adapt their production based on previous experience—specifically, based on task-relevant errors—, and that information transfer is an important task-relevant dimension.

## Footnotes

^1^The stronger claim, *efficient* information transfer, is discussed elsewhere (e.g., Levy and Jaeger, 2007; Jaeger, 2010b; Piantadosi et al., 2011).
